# Risks of passive use of social network sites in youth athletes: a moderated mediation analysis

**DOI:** 10.3389/fpsyg.2023.1219190

**Published:** 2023-10-27

**Authors:** Weipeng Zhang, Feng Jiang, Yuanjiao Zhu, Qiang Zhang

**Affiliations:** ^1^School of Sports Sciences, Tianjin Normal University, Tianjin, China; ^2^School of Business Administration, Jeonbuk National University, Jeonju, Republic of Korea; ^3^School of Physical Education, Woosuk University, Jeonju, Republic of Korea; ^4^Department of Sports, Cangzhou Normal University, Cangzhou, China

**Keywords:** passive social network site use, upward social comparison, subjective well-being, anxiety, positive psychological capital

## Abstract

**Background:**

With the popularity of social media platforms, the use of social networks challenges the well-being and mental health of athletes.

**Motivation:**

Despite ongoing scholarly discussions about the effects of passive use of social network sites, few studies have examined the relationship between the passive use of social network sites and mental health in young athletes from a social comparison perspective.

**Hypothesis:**

To address this research gap, we draw on the social comparison and developmental systems theories to explore the mediating effect of upward social comparison on passive social network site use and mental health, as well as the moderating effects of positive psychological capital.

**Methods:**

We analyzed data about 350 young athletes from professional Chinese sports universities.

**Results:**

As predicted, passive use of social network sites by young athletes increased anxiety (β = 0.26, *p* < 0.001) and decreased subjective well-being (β = −0.35, *p* < 0.001). Upward social comparisons had positive (β = 0.22, *p* < 0.001) and negative (β = −0.34, *p* < 0.001) mediating effects in passive social network site use and anxiety/subjective well-being. Positive psychological capital played a moderating effect between upward social comparison and anxiety (β = −0.28, *p* < 0.001), and subjective well-being (β = 0.24, *p* < 0.001); the moderated mediation effect was also supported.

**Conclusion:**

Our study informs the current research by highlighting the importance of upward social comparison as a critical mechanism and positive psychological capital as a boundary condition. We suggest actively maintaining and enhancing positive psychological capital to mitigate the adverse effects of upward social comparison.

## 1. Introduction

Social networking sites are virtual online platforms for individuals to communicate, build, and maintain relationships with others (Grieve et al., 2013). As of 2020, the number of people using social networking sites has reached 3 billion (Musetti et al., 2022). The increasing usage of social network sites worldwide has led many scholars to study the impact of social network use on human mental health (Berryman et al., 2018; Valkenburg et al., 2022). Studies have found positive and negative effects (Krasnova et al., 2015), with the causes depending on whether individuals are passive or active users of social networking sites (Verduyn et al., 2021). Passive social network site use is the behavior of individuals who only view others’ profiles or statuses without leaving any messages or comments. Active social network site use is behaviors that facilitate online information communication (Verduyn et al., 2015). Due to the fact that the majority of time, most users engage in passive use (Verduyn et al., 2022), this passive use occupies an important place in today’s society.

In addition, the popularity of social media platforms has challenged athletes’ physical and mental health (Brougham, 2021), and mental health is an important resource for athletes in relation to their performance and development (Schinke et al., 2018). Therefore, special attention should be paid to the group of young athletes. Previous studies have focused on the effects of passive use of social network sites on college students and adolescents (Cheng et al., 2023; Zhang et al., 2023), ignoring the effects of passive use of social networks on the mental health of young athletes. Considering the stress of young athletes in all aspects including competition, training, and socialization (Hamlin et al., 2019), as well as the diversity of social media and its inherent social attributes that are particularly appealing to the younger generation (Orben, 2020). This makes it more likely for young athletes to get caught up in the ripples of social media, which can lead to mental health concerns. Therefore, it becomes crucial to examine how social networking affects the mental health of young athletes.

To better understand the relationship between passive use of social network sites and young athletes’ mental health, we used upward social comparison as a mediating mechanism between passive use of social network sites and mental health from a social comparison perspective. Social comparison is the comparison of one’s abilities and opinions with those of others (Festinger, 1954). According to social comparison theory, individuals evaluate their emotional states through social comparison when they are in a new emotional state or unable to judge their emotional state using physiological or empirical cues (Suls and Miller, 1977). Given that social networks primarily present embellished information, this increases the likelihood that individuals will make upward social comparisons (Carraturo et al., 2023). The upward social comparison produces a contrast effect, and negative emotions arise when individuals do not perceive their present or future to be as good as that of the comparison population (Jordan et al., 2011). This implies that young athletes who compare themselves to others and perceive themselves as inferior may experience negative emotions and lower self-evaluation. Therefore, this study suggests that young athletes who passively use social networking sites may use upward comparisons to impact psychological well-being (anxiety and subjective well-being).

Notably, the process and outcome of upward social comparison may depend on individual psychological factors. For example, positive psychological capital is a positive psychological state exhibited by individuals during growth and development (Luthans et al., 2005). Individuals with high positive psychological capital are confident and hopeful about their ability to achieve their goals. They can remain optimistic and face bad situations with a positive mindset (Newman et al., 2014), while positive psychological capital shows a significant negative correlation with anxiety and depression (Turliuc and Candel, 2022) and significant positive correlation with subjective well-being (Datu and Valdez, 2016). Therefore, when young athletes have high positive psychological capital, they believe they can reach the comparator level and are hopeful for the future. Thus, they will alleviate the negative emotions caused by upward comparison (Rahimnia et al., 2013). Previous studies have shown that positive psychological capital acts as a moderating variable to buffer the negative impact of stressors on mental health (Darvishmotevali and Ali, 2020). Considering that upward social comparison as a stressor can be stressful (Greenberg et al., 2007), we argue that positive psychological capital can mitigate the negative effects of upward social comparison on mental health. That is, having high positive psychological capital would reduce the positive effects of upward social comparison on anxiety and mitigate its negative impact on subjective well-being in young athletes.

The overall goal of this study was to examine whether passive social network site use negatively affects anxiety and subjective well-being among young athletes and identify the mechanisms involved in this process. Knowing how passive use of social media affects well-being and anxiety and the factors that influence this relationship can help educators teach young athletes appropriate social media use and prevent negative outcomes.

Thus, this study presents three important contributions to the literature and theory. First, we shift the central focus of the literature on passive social network site use from general social network sites users to young athletes. In this way, we reveal the consequences of passive social network site use by young athletes, with practical implications for managing athletes. Second, we investigated the mechanisms mediating the effects of passive social network site use on mental health. This contribution has practical implications for the development of practices and interventions for passive social network site use aimed at curbing the negative effects on athletes’ mental health. Third, our study mitigates the potential negative effects of passive social network site use by identifying positive psychological capital as a potential moderator. Our research has contributed to the understanding of positive psychological capital.

## 2. Theoretical framework and hypotheses

### 2.1. Passive social network site use and mental health

Social networking sites generally refer to web services that allow users to construct a public or semi-public image within a social network (Boyd and Ellison, 2007; Yue et al., 2022). Young athletes who passive use of social networks sites tend to only view content on social media without directly interacting with content providers (Krasnova et al., 2013; Frison and Eggermont, 2016). Previous research has found that passive social network site use decreases the experience of positive emotions and leads users to experience more negative emotions (e.g., depression, Chhatwani et al., 2023), and further reduce subjective well-being (Verduyn et al., 2017). Furthermore, experimental studies on individuals who use social network sites found that participants who passively used social network sites showed a significant decline in mental health (Cheng and Nhan, 2021). Therefore, we hypothesize the following:

*Hypothesis 1*: Passive social network site use by young athletes will (a) increase anxiety and (b) decrease subjective well-being.

### 2.2. Mediation effects of upward social comparison

Most prior studies have focused on the direct effect of passive use of social network sites on mental health, with little known about the mediating mechanisms and moderating factors in this relationship (Chen et al., 2016). Thus, we used social comparison theory to describe the mediating role of upward social comparison in the relationship between passive use of social network sites and mental health, and developmental systems theory to describe the moderating mechanisms of positive psychological capital.

Festinger (1954) believed that social comparison internally drives individuals to evaluate their perceptions and abilities and promotes comparison with others; he proposed a “theory of social comparison” based on these ideas. The social comparison theory suggests that individuals tend to compare themselves with people in better situations for self-development purposes. However, upward social comparisons are prone to contrasting effects (Gerber et al., 2018). The expectation that one’s future will differ from the comparison goal, the individual will lower their self-evaluation level, leading to a negative impact on them (Xing and Yu, 2006).

Social network sites provide a large amount of information about others and have become a new channel for modern people to obtain information and make social comparisons with others (Escobar-Viera et al., 2018; Burnell et al., 2019; Reer et al., 2019). Information about others on social networking sites plays an important role in promoting social comparison, a phenomenon also supported by social comparison theory (Mussweiler et al., 2006). First, individuals tend to present a positive image of themselves, and users display positive images more often than negative ones on social networking platforms, the information posted on social network sites is always overly positive (Li, 2018). Therefore, young athletes are more likely to see this positive information and make upward social comparisons with this positive information. Second, according to social comparison theory, for self-development purposes, young athletes tend to make upward social comparisons with their relative success. In addition, the overload of information provided on social networks tends to lead to social network fatigue, with increasing numbers of users just browsing rather than posting and sharing (Zhang et al., 2016), resulting in passive social network use being the most common social network behavior (Zheng et al., 2020). Prior research has also found that college students who passively use social networks are more likely to engage in upward social comparisons (Pang, 2021). Therefore, this study hypothesized that passive social network site use by young athletes is more likely to form upward social comparisons in social networks.

When young athletes make upward social comparisons with others on social networking sites, they develop perceptions such as “they are better than me” and “they have a better life than I do.” This increases negative personal emotions (Chou and Edge, 2012) and decreases subjective happiness. That is when sports college students compare themselves with people who are better than them on social networking sites, it leads to a contrast effect of self-evaluation and a feeling that they are inferior to others, negatively impacting the individual. Based on the above discussion and theory, this study proposes the following hypothesis:

*Hypothesis 2*: Upward social comparison mediates the relationship between young athletes’ passive social network site use and (a) subjective well-being and (b) anxiety.

### 2.3. Positive psychological capital as a moderator

Developmental systems theories, which provide a model of individual-environment interactions, suggest that individual psychological developmental outcomes are shaped and developed by interacting environmental and individual factors (Lerner et al., 2006). Therefore, this study introduces the personal aspect of positive psychological capital to expect to reduce the negative effects of upward social comparison on individual mental health.

Positive psychological capital refers to the positive psychological states that individuals exhibit during their growth and development. It is a psychological resource that promotes personal growth and includes self-efficacy, hope, optimism, and resilience (Luthans and Youssef, 2004; Luthans et al., 2007). Individuals with high positive psychological capital believe they can achieve their goals, have hope for the future, have more positive emotions (Avey et al., 2008), and have lower stress and anxiety (Avey et al., 2009). Further, positive psychological capital positively impacts life satisfaction (Karatepe and Talebzadeh, 2016) and subjective well-being (Datu and Valdez, 2016).

According to the social comparison theory, individual expectations can play a decisive role in the outcome of upward comparisons (Suls and Wheeler, 2000). When young athletes have high positive psychological capital, comparative outcomes are more likely to be in the expected range. They may be less likely to feel anxiety due to upward comparisons. Young athletes with high levels of positive psychological capital believe in their ability to close the current gap, believe they can reach the same level as the person being compared, and mitigate the negative effects of upward social comparison on subjective well-being. Conversely, young athletes with low levels of positive psychological capital believe that they have limited ability to reach the level of individuals being compared. Moreover, they have a negative mindset about the future and believe they cannot live as well as these individuals do and therefore feel more anxiety due to upward comparison. Previous research has also demonstrated the moderating effect of positive psychological capital on the relationship between negative factors and mental health. Xiong et al. (2020) concluded that positive psychological capital could mitigate the negative effects of cumulative risk on anxiety and depression in secondary school students. Additionally, Gupta and Bakhshi (2018) demonstrated that positive psychological capital mitigates the negative effects of workplace bullying on subjective well-being. Therefore, we hypothesize the following:

*Hypothesis 3*: Positive psychological capital (a) negatively moderates the relationship between upward social comparison and anxiety and (b) positively moderates the relationship between upward social comparison and subjective well-being.

We proposed that the relationship between passive social network site use and anxiety and well-being in young athletes is moderated by their level of positive psychological capital and that this relationship is mediated by upward social comparison. Specifically, young athletes with higher positive psychological capital levels weaken the positive impact of passive social network site use on anxiety through upward social comparison and alleviate the negative impact on subjective well-being. Lower positive psychological capital reinforces the positive effect of the passive use of social networking sites on anxiety through upward social comparison and a negative effect on subjective well-being. Therefore, we hypothesize the following:

*Hypothesis 4*: The indirect effects of passive social network site use via upward social comparison on (a) anxiety is negatively moderated by positive psychological capital and (b) subjective well-being is positively moderated by positive psychological capital.

In summary, to test the above hypotheses, we chose the quantitative analysis method. This approach allows for simultaneous consideration of the relationships between multiple variables and in-depth exploration of the impact mechanisms between variables (e.g., the moderating effect of positive psychological capital). Moreover, the results of the study can be quantified, which is important for applying the findings to practice and developing interventions.

## 3. Materials and methods

### 3.1. Participants and procedures

This study used a questionnaire that included basic information on passive or active social network use, upward social comparison, anxiety, subjective well-being, positive psychological capital, and demographics. We created the questionnaire through Credamo, a professional data collection platform in China, and recruited young athletes in China through the snowball sampling method with the help of friends from sports schools. Researchers sent the survey link to each participant via WeChat, the most popular social media platform in China. A valid final sample of 350 participants was obtained, and each participant received a cash prize. Among the valid participants, 156 (44.6%) were women and 194 (55.4%) were men, with an age range of 15–17 years (*M* = 16.07; SD = 0.7).

All subjects participated voluntarily and were informed that the survey was anonymous and confidential. The study obtained the electrical consent of all subjects. All procedures in this study met the ethical standards of the Chinese Psychological Association and conformed to the 1964 Declaration of Helsinki and subsequent amendments or similar ethical standards. The Ethics Committee of Tianjin Normal University approved the study (2023050601).

### 3.2. Measures

Following Brislin’s (1986) translation and reverse translation procedures, we translated all scales used in this study from English to Mandarin.

#### 3.2.1. Passive social network site use

The passive social network site use subscale of the Surveillance Use Scale developed by Tandoc et al. (2015) was selected to measure the intensity of individual passive social network site use. The scale asks participants to rate the frequency of social network use behaviors described by four items (e.g., “browsing aggregated dynamic information”). The scale was scored on a five-point scale, with 1 indicating “never” and 5 indicating “frequently.” Higher scores showed a higher intensity of passive social network site use (Cronbach’s α = 0.82).

#### 3.2.2. Upward social comparison

This measure was gaged with two items by adapting from upward social comparison rating scale (De Vries and Kühne, 2015), which uses a question with two possible responses to measure participants’ tendency to compare socially upward online: “When I read news feeds (or see pictures of others), I often think: (1) Other people have a better life than I do; (2) Other people are doing better than I am.” Participants were rated using a 5-point Likert scale, with 1 = very non-conforming and 5 = very conforming, with higher scores indicating a higher degree of upward social comparison. This scale has shown good validity and reliability in previous research (Pang, 2021), and it also showed good reliability in our study (Cronbach’s α = 0.85).

#### 3.2.3. Positive psychological capital

The Positive Psychological Capital Scale by Dudasova et al. (2021) was used to measure positive psychological capital. It is a second-order factor comprised of four items: hope, self-efficacy, optimism, and resilience. An example item is: “I can think of many ways to achieve my current goals.” Participants used a 5-point Likert scale, 1 = very non-conforming and 5 = very conforming, with higher scores indicating higher levels of positive psychological capital. Prior research has demonstrated good internal consistency (Ikeda et al., 2023), and it also had excellent reliability in the present study (Cronbach’s α = 0.92).

#### 3.2.4. Anxiety

The anxiety subscale of the short version of the Depression Anxiety Stress Scale developed by Moussa et al. (2001) was used in this study. Chan et al. (2021) have demonstrated good internal consistency (α = 0.88). The seven items of the scale included sentences describing the individual’s recent (within the last 2 weeks) negative emotions. For example, “I feel like I have lost control and energy during the past 2 weeks.” Participants rated the 7 items using a 5-point Likert scale, with 1 = very non-conforming and 5 = very conforming, with higher scores indicating higher anxiety. Participants made judgments based on the degree of conformity of each item to their own situation (Cronbach’s α = 0.88).

#### 3.2.5. Subjective well-being

The subjective well-being of young athletes was assessed using the World Health Organization Happiness Indicator Scale (Topp et al., 2015), which contains five questions and has shown good validity and reliability in previous research (Dawel et al., 2020). Reasonable judgment on each question was based on how they felt during the past 2 weeks (e.g., I have felt cheerful and in good spirits during the past 2 weeks). Participants were rated using a 5-point Likert scale, with 1 = very non-conforming and 5 = very conforming, with higher scores indicating a higher degree of subjective well-being (Cronbach’s α = 0.88).

#### 3.2.6. Control variables

We controlled for demographic data of young athletes, such as sex and age, and these variables were found to be associated with mental health. For example, Kiely et al. (2019) found that females have poorer mental health than males and De-Juanas et al. (2020) showed that older groups had higher levels of mental health than younger groups. Previous research has shown that active social network site use leads to increased upward social comparison and negatively impacts psychological well-being (Weiser, 2001). This measure was gauged with four items by adapting from active social network site use rating scale (Tandoc et al., 2015), for example, “Update your status.” The scale was scored on a five-point scale, with 1 indicating “never” and 5 indicating “frequently.” Higher scores showed a higher intensity of active social network site use” (Cronbach’s α = 0.83).

#### 3.2.7. Data analytic strategy

Mplus 8.3 (Muthén and Muthén, 2017) was used as a statistical analysis tool to test the model. This is because Mplus provides the user with a comprehensive statistical toolbox for analyzing a wide range of models, and most complicated models can be analyzed with just a few Mplus commands (Javadizadeh, 2020). First, this study performed a confirmatory factor analysis (CFA) using Mplus. CFA goodness-of-fit indices were generally CFI and TLI > 0.90 (Sun, 2005), SRMR <0.08, RMSEA <0.06 (Hu and Bentler, 1999), and χ^2^/df < 3 (Byrne et al., 1989). Second, we assessed the reliability of the scale using Cronbach’s α, as well as the discriminant validity of the scale using Fornell and Larcker's (1981) criteria. We conducted Harman’s one-factor test to test the collected data for common method bias (Podsakoff et al., 2012). Third, we conducted a mediation analysis as suggested by Zhao et al. (2010) and used bootstrapping analysis to examine the mediating effect. This is because bias-corrected confidence intervals provide a more precise assessment of indirect effects than traditional tests of mediation (MacKinnon et al., 2004). This study used 5,000 bootstrap samples, and 95% bias-corrected confidence intervals to test the significance of the indirect effects of upward social comparison. The interval must not contain zero to assume a significant indirect effect (Preacher and Hayes, 2008). Finally, this study employed the moderating effects of high and low levels of positive psychological capital in young athletes, and Mplus was appropriated for moderating analyses and moderated mediation effects.

## 4. Results

### 4.1. Reliability and validity

Before proceeding with the hypothesis, this study conducted confirmatory factor analyses on active or passive social network site use, upward social comparison, positive psychological capital, anxiety, and subjective well-being to assess the fit of the measurement model. The results showed a good fit for the six factors (χ^2^/df = 1.68, CFI = 0.94, TLI = 0.93, RMSEA = 0.04, SRMR = 0.04).

In this study, the reliability analysis is provided on the diagonal of Table 1. The lowest Cronbach’s alpha coefficient for all scales in our study was 0.82. Therefore, the reliability of the scales did not seem to be an issue. In addition, convergent validity was satisfactory as the composite reliability (CR) for each construct ranged from 0.83 to 0.93 (CR > 0.70), and the average variance extracted (AVE) for each construct ranged from 0.51 to 0.74 (AVE > 0.50), both exceeding the suggested thresholds. Discriminant validity was satisfactory as the square root of AVE was greater than for all individual correlations. In addition, the results of Harman’s one-factor test showed that the variance explained by the first rotation factor was 30.73%, less than 50%. Therefore, the questionnaire was not considered to have serious common method bias.

**Table 1:** Descriptive statistics, correlations, and reliabilities.

Variables	*M*	SD	1	2	3	4	5	6	7	8
1. Gender	1.55	0.49								
2. Age	16.07	0.70	0.10							
3. Active social network site use	2.59	0.91	0.05	−0.07	(0.83)					
4. Passive social network site use	3.70	0.52	−0.04	−0.03	−26^**^	(0.82)				
5. Upward social comparison	3.23	1.01	0.03	0.04	−0.20^**^	0.42^**^	(0.85)			
6. Positive psychological capital	4.08	0.45	0.04	0.13^*^	0.04	−0.08	−0.06	(0.92)		
7. Anxiety	2.11	0.57	0.03	0.12^*^	−0.08	0.23^**^	0.36^**^	−0.33^**^	(0.88)	
8. Subjective well-being	3.91	0.90	−0.04	−0.15^**^	0.06	−0.29^**^	−0.40^**^	0.33^**^	−0.54^**^	(0.88)

*N* = 350. SD is the standard deviation. The constructs’ internal reliabilities (alpha coefficients) are given in parentheses on the diagonal. Gender = “female” (1), “male” (2); ^*^p < 0.05. ^**^p < 0.01.

### 4.2. Descriptive statistics and correlation analysis

#### 4.2.1. Descriptive statistics of the scales (mean and standard deviations) and Pearson’s correlation

The coefficients of these variables are listed in Table 1. Passive social network site use was significantly positively correlated with upward social comparison (*r* = 0.42, *p* < 0.01) and anxiety (*r* = 0.23, *p* < 0.01). Further, it was significantly negatively correlated with subjective well-being (*r* = −0.29, *p* < 0.01). Upward social comparison was significantly positively correlated with anxiety (*r* = 0.36, *p* < 0.01) and significantly negatively associated with subjective well-being (*r* = −0.40, *p* < 0.01). Positive psychological capital was significantly negatively correlated with anxiety (*r* = −0.33, *p* < 0.01) and significantly positively associated with subjective well-being (*r* = 0.33, *p* < 0.01). Anxiety and subjective well-being were significantly negatively correlated (*r* = −0.54, *p* < 0.01).

### 4.3. Hypothesis testing

**Figure 1:**
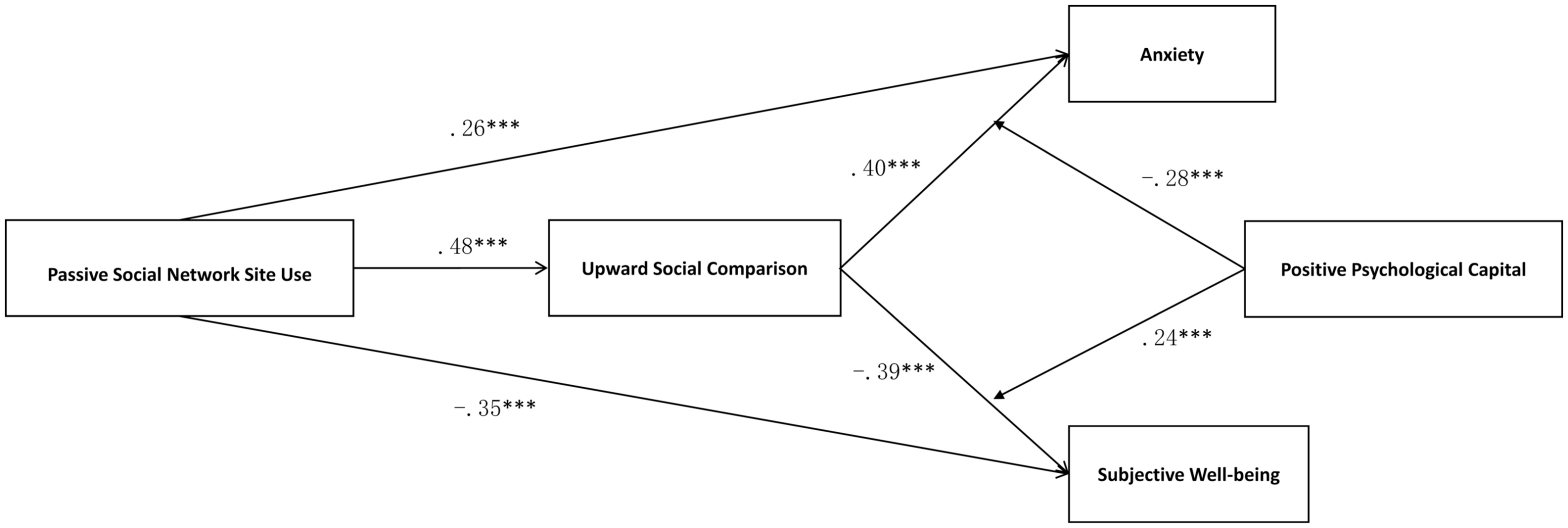
Mediation intermediary model.

**Figure 2:**
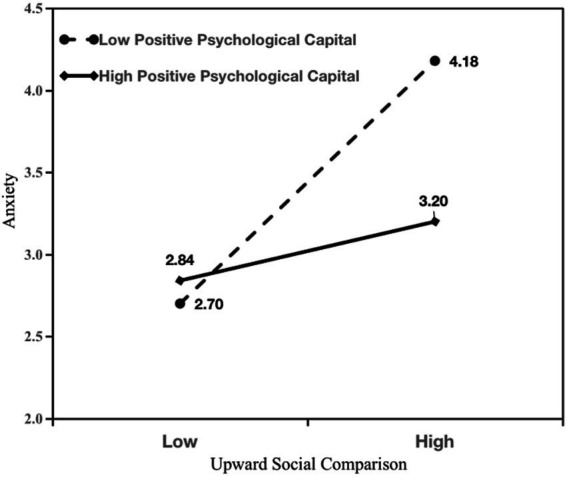
Interaction of upward social comparison and positive psychological capital on anxiety.

**Figure 3:**
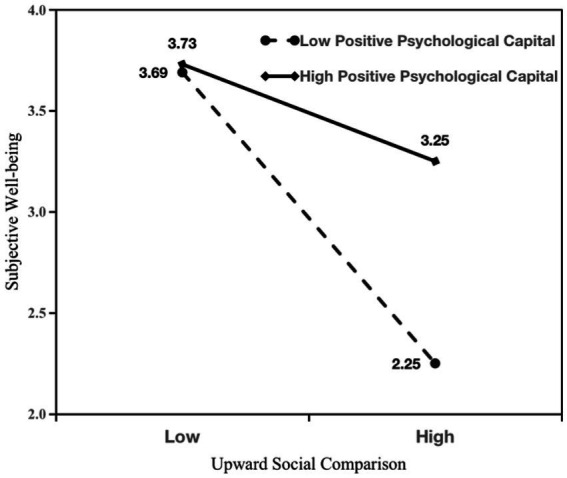
Interaction of upward social comparison and positive psychological capital on subjective well-being.

The results of the hypothesis test are shown in Table 2; Figure 1. In support of hypotheses 1a and 1b, the results of the analysis showed that passive social network site use was positively associated with anxiety (β = 0.26, *p* < 0.001; see Model 2) and subjective well-being (β = −0.35, *p* < 0.001; see Model 5). As predicted, the results of Models 1 and 3 showed that the positive indirect effect of passive social network site use on anxiety via upward social comparison (β = 0.22, *p* < 0.001, 95% C.I. [0.12, 0.34]) was significant, indicating a complementary mediating effect (Zhao et al., 2010). The results of Models 1 and 6 showed that the negative impact of passive social network site use on subjective well-being through upward social comparison (β = −0.34, *p* < 0.001, 95% C.I. [−0.55, −0.21]) was significant, indicating a complementary mediation effect (Zhao et al., 2010). In support of hypotheses 3a and 3b, we examined the effects of upward social comparison on anxiety and subjective well-being at high and low levels of positive psychological capital. The analysis revealed that the interaction between upward social comparison and positive psychological capital significantly predicted anxiety (β = −0.28, *p* < 0.001; see Model 4) and subjective well-being (β = 0.24, *p* < 0.001; see Model 7). The interaction effects are plotted in Figures 2, 3. Simple slope analysis indicated a stronger relationship between upward social comparison on anxiety (β = 0.43, *p* < 0.001) and a stronger negative relationship between subjective well-being (β = −0.64, *p* < 0.001) when young athletes’ positive psychological capital was low. Moreover, a weaker relationship between upward social comparison on anxiety (β = 0.10, *p* < 0.05) and a weaker negative relationship between subjective well-being (β = −0.21, *p* < 0.01) when young athletes’ positive psychological capital was high. In support of hypotheses 4a and 4b, the conditional indirect effects of passive social network site use on anxiety and subjective well-being were examined under three positive psychological capital conditions (mean, −1 SD below the mean, and + 1 SD above the mean). As shown in Table 2, the index of moderated mediating effect was significant for anxiety (index = −0.15, SE = 0.07, LLCI, ULCI [−0.31, −0.03]) and subjective well-being (index = 0.24, SE = 0.11, LLCI ULCI [0.04, 0.49]), which showed that a moderated mediation effect did exist.

**Table 2:** The mediating role of upward social comparison.

Dependent variables	Upward social comparison		Anxiety						Subjective well-being					
	Model 1		Model 2		Model 3		Model 4		Model 5		Model 6		Model 7	
Measure	β (SE)	*p*	β (SE)	*p*	β (SE)	*p*	β (SE)	*p*	β (SE)	*p*	β (SE)	*p*	β (SE)	*p*
1. Gender	0.05 (0.05)	0.349	0.05 (0.06)	0.411	−0.03 (0.05)	0.642	−0.01 (0.05)	0.855	0.04 (0.05)	0.506	−0.05 (0.05)	0.353	0.01 (0.05)	0.940
2. Age	−0.04 (0.05)	0.497	0.08 (0.06)	0.169	−0.06 (0.05)	0.230	−0.03 (0.05)	0.577	−0.14 (0.05)	0.009	−0.03 (0.05)	0.533	−0.09 (0.05)	0.079
3. Active social network site use	−0.07 (0.06)	0.273	0.02 (0.07)	0.800	0.05 (0.05)	0.364	0.05 (0.06)	0.397	−0.03 (0.06)	0.630	−0.06 (0.05)	0.252	0.03 (0.05)	0.571
4. Passive social network site use	0.48 (0.06)	0.000	0.26 (0.06)	0.000	0.07 (0.08)	0.409			−0.35 (0.06)	0.000	−0.16 (0.06)	0.013		
5. Upward social comparison					0.40 (0.07)	0.000	0.46 (0.05)	0.000			−0.39 (0.06)	0.000	−0.48 (0.05)	0.000
6. Positive psychological capital							−0.21 (0.06)	0.001					0.26 (0.06)	0.000
*Interaction							−0.28 (0.06)	0.000					0.24 (0.06)	0.000
*R* ^2^	0.26		0.07		0.19		0.35		0.14		0.25		0.40	
*ΔR* ^2^	0.25		0.06		0.18		0.34		0.13		0.24		0.39	
*F*	30.30^**^		6.49^***^		16.14^**^		30.78^**^		14.04^**^		22.93^**^		38.11^**^	
Index of moderated mediation			Index (SE) LLCI ULCI						Index (SE) LLCI ULCI					
Positive psychological capital			−0.15 (0.07) −0.31 –0.03						0.24(0.11) 0.04 0.49					

*N* = 350. β is the standardized regression coefficient, and SE is the standard error. Bootstrap sample size = 5,000. Gender = “male” (1), “female” (2); *Interaction = Upward Social Comparison × Positive Psychological Capital. ^**^p < 0.01, ^***^p < 0.001.

Although the results were significant for all three values, differences were evident in the results regarding the indirect effects. Specifically, the conditional indirect effect of passive social network use on anxiety (β = 0.07, 95% CI = [0.01, 0.13]) was lower when positive psychological capital was high (+1 SD above the mean) than when positive psychological capital was low (−1 SD below the mean) on anxiety (β = 0.20, 95% CI = [0.11, 0.32]). Similarly, the conditional indirect effect on subjective well-being when positive psychological capital was high (β = −0.12, 95% CI = [−0.25, −0.01]) was lower than the conditional indirect effect on subjective well-being when positive psychological capital was low (β = −0.33, 95% CI = [−0.51, −0.20]).

## 5. Discussion

Despite considerable interest in social media platforms, relatively little is known about how passive social network site use is linked to the mental health and well-being of young athletes in competitive sports. This study constructed a conceptual research model. The model was based on the social comparison theory to understand the effects of passive social network site use on anxiety and subjective well-being, mediating effect of upward social comparison, and moderating effect of positive psychological capital on young athletes. The study showed a significant effect of passive social network site use on upward social comparison, anxiety, and subjective well-being. Furthermore, positive psychological capital also significantly modified upward social comparison, anxiety, and subjective well-being. These findings make a new contribution to the literature by providing results on passive social network site use that contribute to understanding the critical mechanistic role of passive social network site use in predicting subjective well-being and anxiety in young athletes.

### 5.1. Theoretical implications

This study contributes to the literature in several ways. First, our findings suggest that passive social network site use and young athletes’ anxiety and subjective well-being had significant effects. This study suggests that the more young athletes passively use social networks, the more it predicts poor mental health and lower levels of well-being. Our study supports previous findings that young athletes who use social networking sites passively experience more anxiety (O’Day and Heimberg, 2021) and lower subjective well-being (Ding et al., 2017). Verduyn et al. (2021) showed that passive social network site use is negatively associated with mental health. Previous studies related to the effects of passive social network site use on mental health have focused on college students or social network sites users in general (Chen et al., 2016; Wang et al., 2017), and our study focusing on young athletes was able to fill a gap in this research area. Young athletes are a special group of people who are subjected to unique psychological pressures and challenges in sports training and competition. Examining the mental health effects of young athletes’ passive social network site use provides a new perspective on promoting the mental health of young athletes.

Second, our study supports previous findings that passive social network site use leads to upward social comparison (Hu and Liu, 2020), while upward social comparison affects anxiety (Zheng et al., 2020) and subjective well-being (Morry et al., 2018). Our study found that upward social comparison is an essential potential mediating mechanism between passive social network site use and anxiety and subjective well-being. This result not only provides a more in-depth interpretation of previous research, but also further emphasizes the pathways through which passive social networking site use influences anxiety and subjective well-being in young athletes, extending previous research. This finding advances our understanding of how passive social network site use predicts the mental health of young athletes.

Third, we tested whether positive psychological capital in young athletes moderated the relationship between upward social comparison, anxiety, and subjective well-being. The results indicate that higher positive psychological capital mitigates the negative effect of upward social comparison on subjective well-being and weakens the positive effect of upward social comparison on anxiety compared to lower positive psychological capital. This is in line with Darvishmotevali and Ali (2020), who found that the impact of insecurity on a person’s subjective well-being varies according to their positive psychological capital. This consistent result reinforces the importance of positive psychological capital in the field of mental health and further supports our theoretical framework. A review of the literature shows that researchers have made efforts to determine the factors that have the potential to reduce the negative consequences of upward social comparison (Wang et al., 2017; Pang, 2021), but interactions with upward social comparisons to improve mental health have been less studied (Chen et al., 2016). Thus, this study remedies the lack of research on the boundary conditions between the passive use of social networks and mental health and has the potential to contribute to the literature on research variables.

Fourth, we found that the indirect effect of passive social network use on mental health via upward social comparison varies according to the level of positive psychological capital. Specifically, the higher the athlete’s positive psychological capital, the weaker the indirect effect of passive social network use on mental health via upward social comparison would be. Our results from this study strengthen our understanding of the study variables in the above relationships by examining the buffering effect of positive psychological capital. It also enhances the potential of interventions to improve subjective well-being and reduce anxiety in young athletes.

### 5.2. Practical implications

Our results, based on a large sample of young athletes, provide a general picture of what this relationship might look like and have important practical implications for guiding and promoting the development of mental health in young athletes. First, passive social network site use has a direct negative impact on the mental health of young athletes. Most social media users may not be aware of the negative effects of passive social network site use on individuals, nor do they necessarily respond productively (Fox and Moreland, 2015). Therefore, young athletes must recognize that passive social network site use can negatively impact an individual’s mental health and reduce passive social network site use, do not frequently “browse WeiBo” and “browse WeChat circles,” and reduce the passive acceptance of other people’s posts showing their lives.

Second, our findings suggest that young athletes who passively use social network sites are more likely to engage in upward social comparisons that impair mental health. In order to better manage the mental health of young athletes, coaches can tell them that the information on social network sites is carefully processed by others and not to pay too much attention to it. And coaches can encourage them to focus on their strengths and accomplishments (Hobfoll, 2001) and find their own values.

Finally, this study showed that young athletes with high positive psychological capital had lower anxiety and higher subjective well-being after an upward social comparison. Therefore, it is necessary for coaches to focus on the positive inner resources of athletes and develop practical strategies to enhance the positive psychological capital of young athletes to improve their mental health. For example, young athletes can be familiarized with team goals and informed of their significant contributions in achieving them, informed of their positive traits and competencies, and guided on how to create and enhance positive internal attributes.

### 5.3. Limitations and future recommendations

The study found significant relationships between passive social media use, upward social comparison, and positive psychological capital on mental health in athletes. However, the study’s design was cross-sectional, meaning that data were collected at only one point in time and from self-reported experiences of anxiety and well-being. This does not allow for definitive conclusions regarding causal relationships between these variables. In addition, since we used quantitative analysis, we were unable to delve into the reasons for such results. Future research could use experimental or longitudinal studies to consider relationships between variables. For example, the type of social networking use (active vs. passive) and the level of social networking use (light vs. heavy) could be manipulated to measure the impact of passive use of social network sites on mental health.

Second, this study only distinguished between active and passive use and explored the impact of social networks on mental health from the perspective of passive use. Future research could analyze passive use from an economic cost–benefit perspective. For example, one could consider the differences in time input, resource input, and attentional input of passive use of social network sites on the mental health of young athletes. This contributes to the understanding of the economic costs and benefits of passive use of social network sites on the mental health of athletes.

Third, we only tested the moderating role of positive psychological capital as an intrinsic personal resource and the mediating role of upward social comparison without discussing other potential mechanisms. Indeed, anxiety as a negative emotion (Thorisdottir et al., 2019) and emotion regulation and management substantially impact mood (Tamminen et al., 2021). Therefore, future research should examine the role of emotion regulation in mediating upward social comparison.

Finally, although sufficiently convincing, the sample size was small and not generalizable because we only collected data from young athletes and not from athletes of different age groups. Therefore, future studies should collect data from a broader range of athletes.

## 6. Conclusion

Based on social comparison theory, this study explored the mechanisms of young athletes’ passive use of social network sites on their mental health. Previous studies have found a relationship between the passive use of social network sites and mental health (Yue et al., 2022). Still, there has been limited research on the mediating and boundary mechanisms between the passive use of social network sites and mental health (Chen et al., 2016), especially in a specific group of young athletes. The results showed that passive use of social network sites has a direct negative impact on the mental health of young athletes. Passive use of social network sites also indirectly affects mental health through upward social comparison. Furthermore, as an essential mechanism, positive psychological capital plays a vital role in improving the mental health of young athletes.

## Data availability statement

The raw data supporting the conclusions of this article will be made available by the authors, without undue reservation.

## Ethics statement

The studies involving humans were approved by Ethics Committee of Tianjin Normal University. The studies were conducted in accordance with the local legislation and institutional requirements. Written informed consent to participate in this study was not required from the participants in accordance with the national legislation and the institutional requirements.

## Author contributions

WZ designed and drafted the manuscript. FJ and YZ designed the method and drafted the initial manuscript. QZ collected the data. All authors contributed to the article and approved the submitted version.
