# Supplementary material for: Risks of passive use of social network sites in youth athletes: a moderated mediation analysis
Source: Front Psychol. 2023 Oct 27;14:1219190. doi: 10.3389/fpsyg.2023.1219190 (PMC10641791; doi:10.3389/fpsyg.2023.1219190)
Supplement: Supplementary file 1 [file Data_Sheet_1.pdf]

Supplementary Material: Questionnaire

Items of the Variables

All questions were scored on a five-point Likert scale ranging from 1 (very non-conforming) to 5 (very conforming).

- *Passive Social Network Site Use (PSNSU)*

PSNSU1: Read the 'newsfeed'.

PSNSU2: Read a friend's status update.

PSNSU3: View a friend's photo.

PSNSU4: Browse a friend's timeline.

- *Active Social Network Site Use (ASNSU)*

ASNSU1: Write a status update.

ASNSU2: Post your photos.

ASNSU3: Comment on a friend's post.

ASNSU4: Proactive chatting with friends.

- *Upward Social Comparison (USC)*

When I browse news feeds or look at photos of other people, I often think that:

USC1: Other people have more better living conditions than I do.

USC2: Other people are doing better than I am.

- *Positive Psychological Capital (PsyCap)*

PsyCap1: If I should find myself in a jam, I could think of many ways to get out of it

PsyCap2: Right now, I see myself as being pretty successful.

PsyCap3: I can think of many ways to reach my current goals.

PsyCap4: I am looking forward to the life ahead of me.

PsyCap5: The future holds a lot of good in store for me.

PsyCap6: Overall, I expect more good things to happen to me than bad.

PsyCap7: I consider myself to be able to stand a lot, I am not easily discouraged by failure.

PsyCap8: After serious life difficulties, I tend to quickly bounce back.

PsyCap9: I believe that coping with stress can strengthen me.

PsyCap10: I am confident that I could deal efficiently with unexpected events.

PsyCap11: I can solve most problems if I invest the necessary effort.

PsyCap12: I can remain calm when facing difficulties because I can rely on my coping abilities.

- *Anxiety*

Participants rate the extent to which they have experienced each item over the past week：

Anxiety1: Dryness of mouth.

Anxiety2: Breathing difficulty.

Anxiety3: Trembling.

Anxiety4: Worried situations panic.

Anxiety5: Close to panic.

Anxiety6: Aware action heart.

Anxiety7: Scared no good reason.

- *Subjective Well-being (SWB)*

Participants rate how well each of the 5 statements applies to him or her when considering the last 14 days：

SWB1: I have felt cheerful and in good spirits.

SWB2: I have felt calm and relaxed.

SWB3: I have felt active and vigorous.

SWB4: I woke up feeling fresh and rested.

SWB5: My daily life has been filled with things that interest me.
